# Improved femoral component rotation in advanced genu valgum deformity using computer-assisted measured resection total knee arthroplasty

**DOI:** 10.1186/s13018-015-0279-4

**Published:** 2015-09-02

**Authors:** Shih-Jie Lin, Chien-Ying Lee, Kuo-Chin Huang, Kuo-Ti Peng, Tsan-Wen Huang, Mel S. Lee, Robert Wen-Wei Hsu, Wun-Jer Shen

**Affiliations:** Department of Orthopaedic Surgery, Chang Gung Memorial Hospital, Chiayi, 6, West Section, Chia-Pu Road, Pu-Tz City, 613 Chia-Yi Hsien Taiwan; Department of Orthopaedic Surgery, Kaohsiung Chang Gung Memorial Hospital, No. 123, DAPI Rd. Niaosng Dist., Kaohsiung City, 83301 Taiwan; Chang Gung University, Taoyuan, Taiwan, 259 Wen-Hwa 1st Road, Kwei-Shan Tao-Yuan, 333 Taiwan; Po-Cheng Orthopedic Institute, 100 Bo-ai, 2nd Road, Zuoying District, Kaohsiung Taiwan

**Keywords:** Genu varus, Genu valgus, Rotational alignment, Computer-navigated, Total knee arthroplasty

## Abstract

**Background:**

Accurate femoral rotational alignment is of vital importance for successful total knee arthroplasty (TKA). The value of computer-assisted surgery TKA (CAS-TKA) in increasing the accuracy of femoral rotational alignment remains controversial. We hypothesize that outcomes are related to the severity of preoperative varus and valgus deformity and that CAS-TKA may be beneficial under certain circumstances.

**Methods:**

Between January 2007 and December 2013, patients with osteoarthritis and varus angulation in the mechanical axis (MA) ≥ 15° and valgus angulation in the MA ≥ 10° (based on hip-to-ankle standing radiography) who underwent TKA were divided into four groups. CAS-TKA and conventional TKA outcomes were compared in patients who had preoperative advanced genu varum and advanced genu valgum deformities. The accuracy of component alignment and postoperative limb alignment was determined using radiographic parameters and computed tomography (CT).

**Results:**

One hundred and eight patients (144 knees) were included in the analysis. For patients with preoperative advanced genu varum deformity, a significant difference was detected in the sagittal femoral angle (*p* < 0.001), but no significant improvement of femoral rotational alignment was noted (*p* = 0.127). In patients with preoperative advanced genu valgum deformity, a significant difference was found in the sagittal femoral angle (*p* = 0.034). The femoral rotational angle was significantly closer to the proper position in the CAS-TKA group (*p* < 0.001). When comparing the percentage of knees achieving the proper alignment, there was a decrease in the amount of outlier for the femoral rotational angle for CAS-TKA in advanced genu valgum deformity (*p* = 0.011).

**Conclusions:**

Our data demonstrate that CAS-TKA is beneficial in obtaining proper femoral rotational alignment in patients with advanced genu valgum deformity (preoperative MA ≥ 10° valgus). In patients with advanced genu varum deformity (preoperative MA ≥ 15° varus), CAS-TKA did not improve the femoral rotational alignment.

## Introduction

Total knee arthroplasty (TKA) can reduce pain and restore proper function in patients with osteoarthritis [1]. Recent studies suggest that proper femoral rotational alignment correlates with faster rehabilitation, better knee function, and improvement in quality of life, as well as longevity [2–5]. Malrotation of the femoral component not only affects patellar tracking but also contributes to flexion instability, stiffness, abnormal gait patterns, and knee pain [6–9]. Finite element modeling (FEM) and biomechanical study have further confirmed that malrotation of the femoral component increases the contact pressure and leads to excessive wear of the patellar button, as well as premature mechanical loosening of the components [10–12].

Computer-assisted surgery TKA (CAS-TKA) has been used for over 10 years and is considered by some to be a valuable adjunct for improving implant placement accuracy and limb alignment [13–17]. However, the effects of CAS-TKA on rotational alignment remain controversial [18–26]. Recently, CAS-TKA has been found to provide more proper femoral rotational alignment and improve clinical outcome at short- and mid-term follow-up [18–22]. Conversely, other studies have reported that CAS-TKA did not provide better outcomes [23–26]. This inconsistency may be partially due to different types (varus or valgus deformity) and severity of preoperative knee deformity seen in the study populations. Substantial variations in femoral anatomy, bone loss, maltracking of the patella, and the soft tissue contracture may be presented in patients with larger varus and valgus deformities [16, 22, 27–33]. These factors may lead to distortion of bony landmarks, which may lead to errors in determining the reference axis for the femoral component rotation and consequently malposition of the components [22, 27–33]. Little has been published regarding the role of CAS-TKA in patients with a preoperative mechanical axis (MA) ≥15° varus and ≥10° valgus. We hypothesize that greater varus or valgus deformity would make conventional TKA difficult and result in malrotation of the femoral component. The purpose of this study was to investigate whether the existence of advanced preoperative knee deformities would enhance the advantages of CAS-TKA via computed tomography (CT) scan assessment.

## Methods

The Institutional Review Board of Chang-Gung Memorial Hospital (100-2752B) approved the retrospective study, and all patients provided the signed informed consent.

The computer databases at our institution were searched for the records of patients with osteoarthritis of the knee who underwent primary TKA between January 2007 and December 2013. Because the cost of CAS was not reimbursed by our national health insurance, the type of surgery was chosen by the patients themselves after a complete explanation of the merits of both techniques. Exclusion criteria were (a) a preoperative stiff knee, (b) a revision prosthesis or a varus-valgus-constrained type of prosthesis was used, (c) extra-articular deformity of the femur or tibia due to previous trauma or surgery and, (d) incomplete medical records. In addition, patients with sclerosis of the diaphyseal femoral or tibial canal resulting from trauma or previous surgery, or retained hardware, were excluded. All radiographic analyses were executed via anteroposterior and lateral radiographs of the knees, and hip-to-ankle standing radiography was taken preoperatively and postoperatively [34]. All limbs underwent CT scanning to evaluate the rotational alignment of the femoral component using the “Perth CT Protocol” [35] at the time of the last follow-up.

**Fig. 1:**
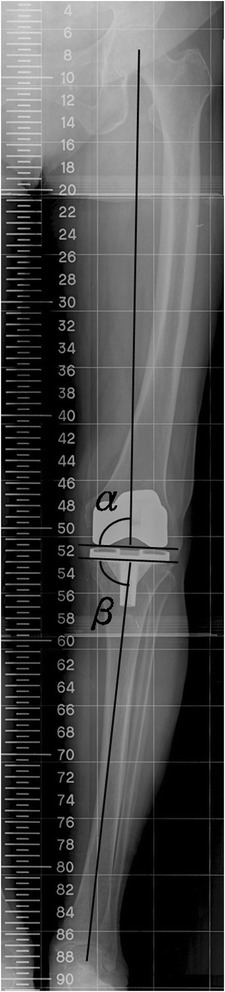
Radiograph showing the measurement of component alignment angles in the coronal plane. The alignment of the femoral components was measured by the intersection of a *line* drawn across the base of the femoral component and the mechanical axis. The alignment of the tibial components was measured by the intersection of a *line* drawn across the base of tibial component and the mechanical axis. Radiographic evaluation system originally appeared in J Bone Joint Surg Am. 2009;91:14–19. *α* frontal femoral angle, *β* frontal tibial angle

**Fig. 2:**
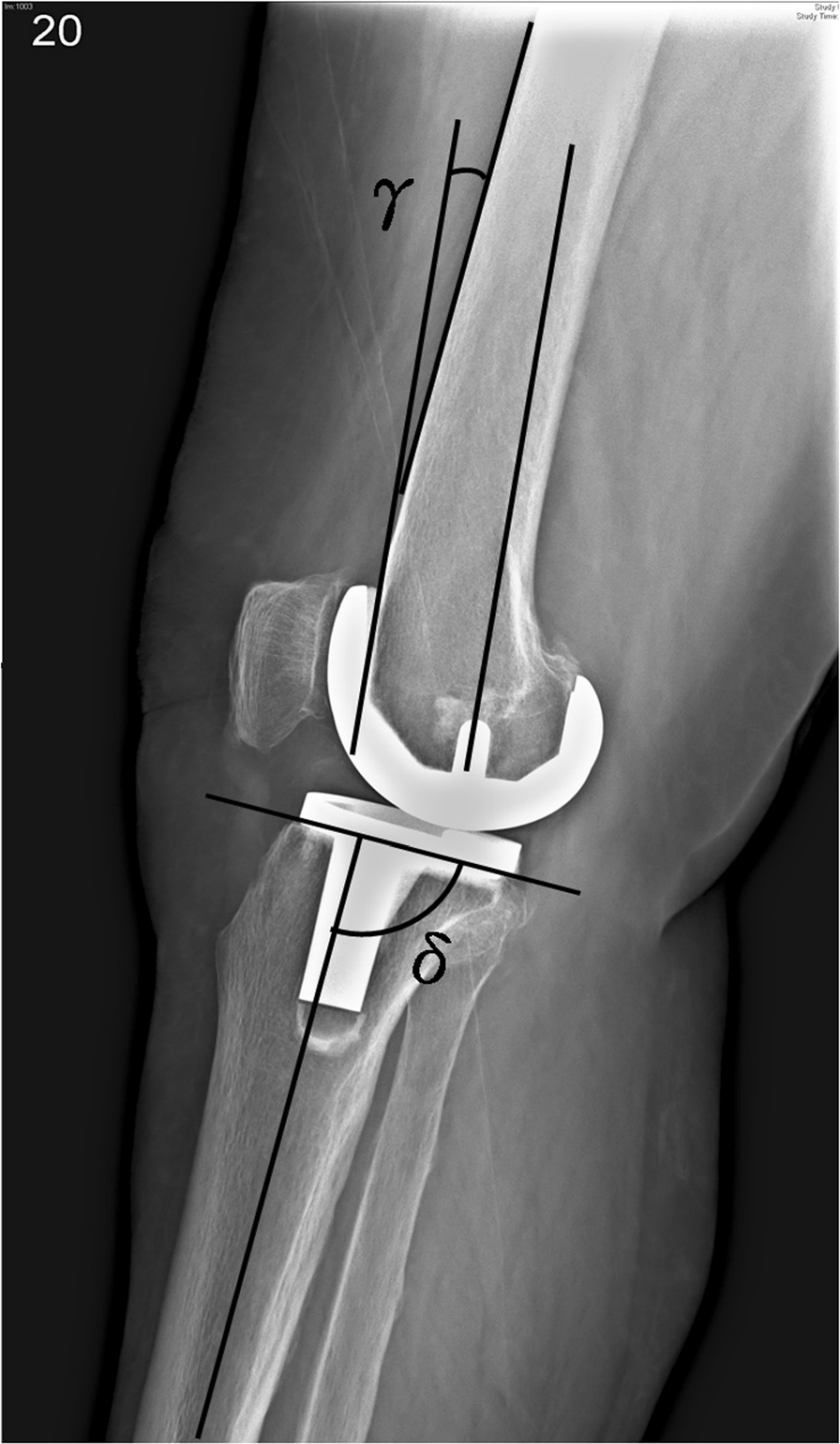
Radiograph showing the measurement of flexion and extension of the femoral component and measurement of the posterior slope of the tibial component in the sagittal plane. Radiographic evaluation system originally appeared in J Bone Joint Surg Am. 2009;91:14–19. *γ* sagittal femoral angle, *δ* sagittal tibial angle

**Fig. 3:**
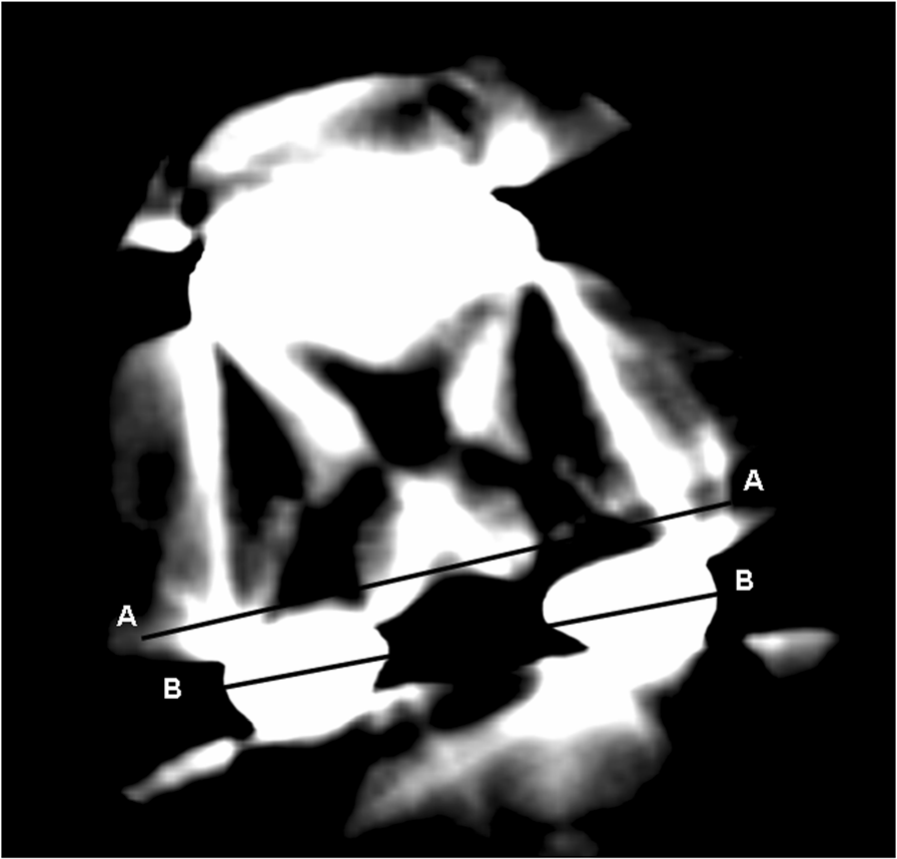
Computed tomography showing measurement of rotation of the femoral component in the axial plane. The femoral rotational (FR) angle was defined as the angle between the surgical epicondylar axis (*AA*) and the tangent to the posterior femoral condyles of the femoral component (*BB*). Measurement of computed tomography originally appeared in J Bone Joint Surg Br. 2004;86:818

Radiographic parameters, including the MA and alignment of components, were measured according to the method described by Kim et al. [26] (Figs. 1 and 2). The following parameters were assessed: MA, frontal femoral (FF) angle, frontal tibial (FT) angle, sagittal femoral (SF) angle, and sagittal tibial (ST) angle. The planned position for the femoral component was a FF angle of 90° in the coronal plane and a SF angle of 0° in the sagittal plane and that of the tibial component was a FT angle of 90° in the coronal plane and an ST angle of 87° in the sagittal plane. The goal was to reconstruct the MA and component alignments to within 3° of the proper position [26, 36]. CT scans allow assessment of the femoral component alignment in the axial plane. According to the Perth CT Protocol [35], the femoral rotational (FR) angle was defined as the angle between the surgical epicondylar axis and the tangent to the posterior femoral condyles of the femoral component (Fig 3). Differences in absolute value from the target angle were recorded and analyzed. The proper femoral rotational angle was defined as within 3° of the target angle (0°) [24, 26, 34, 35]. A blinded observer performed all measurements using digital radiographs and CT on a computer. The intraobserver reliability was assessed, and the intraclass correlation coefficients (ICCs) were measured according to the method described by Konigsberg et al. [37]. The ICCs of the intraobserver reliabilities of all measurements were 0.740 (range 0.681–0.906). Because the measurements were judged reliable, measurements made by this blinded observer were used in the analyses.

### Surgical technique

All TKAs were performed using an anterior midline longitudinal skin incision and a medial parapatellar arthrotomy by a single experienced orthopedic surgeon (RW-WH). The same total knee prostheses (DePuy PFC Knee Systems: DePuy Orthopaedics Inc., Warsaw, IN) were used in all patients. In the conventional group, TKA was performed with the use of an intramedullary alignment guidance system for femoral preparation and an extramedullary guide for tibial preparation. The angle of the cutting block was adjusted according to the valgus correction angle of the distal femur, which was measured on hip-to-ankle standing radiographs. After performing the distal femoral resection, and then exactly identifying the anatomical landmarks, the surgical transepicondylar line was drawn for judgment of femoral rotation. Whiteside’s line, posterior condylar line, and the tibial cutting plane were supplementarily used to judge the femoral rotation. After the osseous cuts of the femur and tibia were completed, the soft tissue balance was assessed by trial reduction and achieved by sequential release of the tight structures in both flexion and extension. The tourniquet was then deflated, and assessments of hemostasis and patellar tracking were performed.

In CAS-TKA, the prostheses were implanted using a CT-free navigation system (BrainLAB, Munich, Germany). Anatomic landmarks were carefully identified and then sequentially registered into the navigation system. The implant size and orientation were determined by dragging the pointer along the bone surface to reconstruct a three-dimensional (3D) bone model. Femoral preparation was performed first, followed by tibial preparation under the guidance of the CT-free navigation system. The femoral component was referenced parallel to the anterior cortex of the distal femur and the transepicondylar line, which were previously registered in the navigation system. After the osseous cuts of the femur and tibia were completed, the soft tissue balance was assessed by the trial reduction and achieved by sequential release of the tight structures in both flexion and extension under the assistance of computer navigation.

Patients with osteoarthritis and varus angulation in the MA ≥ 15° and valgus angulation in the MA ≥ 10° were divided into four groups: group A, advanced genu varum deformity (defined as MA ≥ 15° varus based on hip-to-ankle standing radiography) who underwent CAS-TKA; group B, advanced genu varum deformity who underwent conventional TKA; group C, advanced genu valgum deformity (defined as MA ≥ 10° valgus based on hip-to-ankle standing radiography) who underwent CAS-TKA; group D, advanced genu valgum deformity who underwent conventional TKA.

All data were collected and entered into an Excel spreadsheet (Microsoft, Redmond, WA) by an independent statistician. After being rechecked for missing and illogical data, the data were copied into SPSS version 13.0 (SPSS Inc., Chicago, IL) for statistical analyses. Fisher’s exact probability test was used to compare the quality of implantation, measured against the proper position, between the two techniques. Student’s *t*-test was applied for comparisons of variables including age, body height, body weight, body mass index (BMI), length of hospital stay, tourniquet time, blood loss, and radiographic parameters. An independent statistician who was blinded to the allocation conducted all statistical analyses. Values of *p* < 0.05 were considered to indicate statistical significance.

## Results

A total of 108 patients (144 knees) were included in the study, and there were 39 knees in group A, 51 knees in group B, 26 knees in group C, and 28 knees in group D. The four groups were comparable with respect to demographic characteristics. Group A had significantly less total blood loss, but had a longer tourniquet time, than group B (Table 1). The tourniquet time was similar between group C and group D (Table 2).

**Table 1:** Demographic and operative data of patients with genu varum

	Group A	Group B	
	*n* = 39	*n* = 51	
Parameters	Mean ± SD (min-max)	Mean ± SD (min-max)	*p* value
Age (years)	68.8 ± 5.7 (53–80)	69.6 ± 5.9 (53–82)	0.476
Body height (cm)	152.2 ± 7.6 (138–175)	151.9 ± 7.2 (138–175)	0.844
Body weight (kg)	68.7 ± 10.4 (50–93)	69.0 ± 10.3 (52–83)	0.861
Body mass index (kg/m^2^)	29.6 ± 4.2 (21–39)	29.9 ± 4.4 (20–39)	0.760
Hospital stay (d)	6.4 ± 1.2 (5–8)	6.8 ± 1.3 (5–9)	0.399
Tourniquet time (min)	78.9 ± 18.0 (51–129)	59.3 ± 15.0	<0.001*
Total blood loss (ml)	519 ± 220 (200–905)	698 ± 319 (220–1205)	0.001*

Group A: osteoarthritis with genu varum and underwent computer-assisted surgery total knee arthroplasty (CAS-TKA)

Group B: osteoarthritis with genu varum and underwent conventional TKA

*p* values for between-group comparison were determined by Mann-Whitney *U* test

*Statistically significant (*p* < 0.05)

**Table 2:** Demographic and operative data of patients with genu valgum

	Group C	Group D	
	*n* = 26	*n* = 28	
Parameters	Mean ± SD (min-max)	Mean ± SD (min-max)	*p* value
Age (years)	71.0 ± 7.6 (52–80)	72.1 ± 7.1 (55–81)	0.583
Body height (cm)	156 ± 8.4 (145–172)	154 ± 6.3 (147–170)	0.546
Body weight (kg)	66.2 ± 10.5 (40–81)	64.1 ± 9.0 (48–79)	0.438
Body mass index (kg/m^2^)	27.2 ± 3.2 (20.8–32.1)	26.8 ± 3.4 (21.8–31.1)	0.685
Hospital stay (d)	6.5 ± 1.2 (4–10)	6.6 ± 1.4 (5–10)	0.688
Tourniquet time (min)	72.1 ± 15.7 (50–120)	72.6 ± 22.7 (58–117)	0.969
Total blood loss (ml)	648 ± 243 (230–765)	762 ± 214 (255–890)	0.075

Group C: osteoarthritis with genu valgum and underwent computer-assisted surgery total knee arthroplasty (CAS-TKA)

Group D: osteoarthritis with genu valgum and underwent conventional TKA

*p* values for between-group comparison were determined by Mann-Whitney *U* test

*Statistically significant (*p* < 0.05)

After analyzing the radiographic data in patients with advanced genu varum deformity, we noted that the preoperative and postoperative coronal MA was very similar between groups A and B. With regard to component alignment, there was a significant difference in the SF angle in the sagittal plane (*p* < 0.001) (Table 3). However, similar FF, FR, FT, and ST angles were noted between the two techniques in advanced varus deformity. In patients with advanced genu valgum deformity, there was no difference between group C and group D with respect to the preoperative and postoperative coronal MA. Similar FF, FT, and ST angles were noted between groups C and D. A difference in the sagittal plane was noted in the SF angle (*p* = 0.034). A significant difference of femoral rotational angle was also found between group C and group D (*p* < 0.001) (Table 4).

**Table 3:** Radiographic data of patients with genu varum

	Group A	Group B	
	*n* = 39	*n* = 51	
Parameters	Mean ± SD (min-max)	Mean ± SD (min-max)	*p* value
Preoperative coronal MA (°)	168.7 ± 8.4 (156–169)	168.5 ± 6.9 (145–170)	0.887
Postoperative coronal MA (°)	179.5 ± 1.7 (178–184)	177.9 ± 2.4 (176–181)	0.572
Frontal femoral angle (°)	90.6 ± 1.7 (86–91)	89.7 ± 1.5 (84–91)	0.414
Sagittal femoral angle (°)	1.6 ± 1.2 (0–6)	3.5 ± 2.8 (1–7)	<0.001*
Femoral rotation angle (°)	1.0 ± 0.7 (0–3)	1.7 ± 1.1 (0–6)	0.127
Frontal tibial angle (°)	90.0 ± 0.5 (89–91)	89.9 ± 1.4 (88–91)	0.714
Sagittal tibial angle (°)	87.9 ± 1.8 (84–90)	87.7 ± 2.2 (80–91)	0.518

Group A: osteoarthritis with genu varum and underwent computer-assisted surgery total knee arthroplasty (CAS-TKA)

Group B: osteoarthritis with genu varum and underwent conventional TKA

*p* values for between-group comparison were determined by Mann-Whitney *U* test

*Statistically significant (*p* < 0.05)

**Table 4:** Radiographic data of the patients with genu valgum

	Group C	Group D	
	*n* = 26	*n* = 28	
Parameters	Mean ± SD (min-max)	Mean ± SD (min-max)	*p* value
Preoperative coronal MA (°)	192.9 ± 0.9 (192–196)	194.9 ± 1.6 (191–197)	0.330
Postoperative coronal MA (°)	180.5 ± 1.4 (176–181)	179.9 ± 2.6 (178–184)	0.321
Frontal femoral angle (°)	88.9 ± 2.0 (88–91)	88.6 ± 1.6 (86–90)	0.586
Sagittal femoral angle (°)	2.1 ± 1.3 (0–6)	3.4 ± 2.1 (1–7)	0.034*
Femoral rotation angle (°)	1.0 ± 0.6 (0–3)	2.9 ± 1.2 (0–6)	<0.001*
Frontal tibial angle (°)	90.0 ± 0.3 (89–91)	89.9 ± 0.4 (88–91)	0.149
Sagittal tibial angle (°)	87.1 ± 1.7 (84–90)	87.7 ± 1.8 (80–91)	0.286

Group C: osteoarthritis with genu valgum and underwent computer-assisted surgery total knee arthroplasty (CAS-TKA)

Group D: osteoarthritis with genu valgum and underwent conventional TKA

*p* values for between-group comparison were determined by Mann-Whitney *U* test

*Statistically significant (*p* < 0.05)

When comparing the percentage of knees achieving the proper alignment, no differences were demonstrated between groups A and B with regard to component alignment angles including the FF (*p* = 0.378), FR (*p* = 0.730), FT (*p* = 0.501), and ST angles (*p* = 1.000). The number of outliers in the axial plane was not significantly reduced in the CAS-TKA group (Table 5). For patients with a preoperative genu valgum deformity, a higher percentage of TKAs achieved the proper femoral component position in the axial plane in group C compared with group D (92.3 vs. 75.0 %, respectively, *p* = 0.011). No postoperative differences between the two groups with regard to other component alignment angles including the FF (*p* = 1.000), FT, and ST angles (*p* = 0.604) were noted (Table 6).

**Table 5:** Comparison of postoperative lower limb axes of patients with genu varum (within 3° deviation) and component positioning

	Postoperative positioning within 3° deviation		
	Group A	Group B	*p* value
	*n* = 39 (%)	*n* = 51 (%)	
Coronal mechanical axis within 3° deviation	36 (92.3)	42 (82.3)	0.220
Component positioning			
Frontal femoral angle	35 (89.7)	42 (82.3)	0.378
Femoral rotation angle	36 (92.3)	46 (90.2)	0.730
Frontal tibial angle	39 (100)	49 (96.1)	0.501
Sagittal tibial angle	37 (94.8)	49 (96.1)	1.000

Group A: osteoarthritis with genu varum and underwent computer-assisted surgery total knee arthroplasty (CAS-TKA)

Group B: osteoarthritis with genu varum and underwent conventional TKA

Data are presented as number (%)

*p* values for between-group comparison were determined by chi-square tests

*Statistically significant (*p* < 0.05)

**Table 6:** Comparison of postoperative lower limb axes of patients with genu valgum (within 3° deviation) and component positioning

	Postoperative positioning within 3° deviation		
	Group C	Group D	*p* value
	*n* = 26 (%)	*n* = 28 (%)	
Coronal mechanical axis within 3° deviation	24 (92.3)	23 (82.1 %)	0.423
Component positioning			
Frontal femoral angle	26 (100)	27 (96.4)	1.000
Femoral rotation angle	24 (92.3)	21 (75.0)	0.011*
Frontal tibial angle	26 (100)	28 (100)	-
Sagittal tibial angle	24 (92.3)	27 (96.4)	0.604

Group C: osteoarthritis with genu valgum and underwent computer-assisted surgery total knee arthroplasty (CAS-TKA)

Group D: osteoarthritis with genu valgum and underwent conventional TKA

Data presented as number (%)

*p* values for between-group comparison were determined by chi-square tests

*Statistically significant (*p* < 0.05)

No complications attributable to the placement of pins for the femoral and tibial reference arrays for CAS-TKAs were noted. Pulmonary emboli, deep vein thrombosis, peroneal nerve neurapraxia, or postoperative wound infection was not noted in any patients. No patients exhibited loosening or osteolysis on radiographs or received revision surgery for any reason at the time of the last follow-up.

## Discussion

The key finding in this investigation was that CAS-TKA is beneficial in obtaining proper femoral rotational alignment in patients with a preoperative MA ≥ 10° valgus. In patients with advanced genu varum deformity (MA ≥ 15° varus), CAS-TKA did not improve the rotational alignment.

A femoral component in a suboptimal position will result in uneven load distribution, imbalance of soft tissue, aberrant kinematic behavior, stiffness, flexion instability, and early loosening [10–12, 26]. Recently, more attention has been paid toward the accuracy of femoral rotational alignment. Numerous surgical techniques including the measured resection CAS-TKA [18–26], gap balancing technique [38–42], and patient-specific instrumentation [43, 44] have been developed to obtain proper component position in the axial plane, yet their effect is inconsistent in the literature [18–26, 38–44]. When performing TKA, some favor a gap balancing technique in which the femoral component is positioned parallel to the tibial cutting plane with collateral ligaments equally tensioned to obtain a rectangular flexion gap. However, the effect of the gap balancing technique on femoral rotational alignment is also inconsistent [38–42]. In addition, concerns exist regarding the changes of joint-line position [39, 40] and excessive external rotation of the femoral component [41, 42].

Based on postoperative CT scans, CAS-TKA with use of the CT-free navigation system has been shown by some authors to offer significant improvement in the rotational alignment of the femoral component when compared with conventional TKA [18–22], while the other researchers have found no difference between the techniques [23–26]. We speculate that the discrepancy may be due to different types and severity of preoperative knee deformity seen in the different study populations. Arthritic knees with large varus and valgus deformity with soft tissue and osseous anomalies may have distortion of bony landmarks, making determination of reference axes difficult when using conventional instrumentation [16, 22, 27–33]. These factors may contribute to consequent malrotation of the femoral component [29–33]. To date, there is little information regarding differences in femoral rotational alignment when TKA is performed with a navigated or conventional technique in the presence of advanced preoperative varus and valgus deformity.

In patients with advanced genu varus deformity, this study showed a significant difference of femoral component alignment in the sagittal plane. Kim et al. [36] studied 3048 knees in 1696 patients and concluded that a sagittal femoral angle greater than 3° was associated with a higher risk of component failure. However, difficulty in achieving good sagittal alignment has been claimed, and the true impact of sagittal malalignment has been rarely been studied and not clearly established [26, 32, 36]. Our data showed no difference in the coronal and rotational alignment of the components between the CAS and the conventional group. The percentage of the femoral components implanted within 3° of the proper rotational alignment was 92.3 % in the CAS-TKA group and 90.2 % in the conventional TKA group. The number of outliers was not significantly reduced in the CAS-TKA group. Rotational alignment in the CT-free navigation system primarily depends on the surgeon’s determination of the anatomic landmarks during surgery, which is a similar method to what is done in conventional surgery. However, inadequate identification of the anatomic landmarks has been reported to be as high as 25 % in arthritic knees. Therefore, surgeons cannot completely rely on the accuracy of the image-free navigation system [27, 28, 30]. CT-based navigation may have an advantage for rotational alignment accuracy because it allows accurate preoperative planning on the patient-specific 3D bone model. However, the CT-based navigation system is associated with additional radiation, additional time for preoperative planning, and cost of the preoperative scans. When adequate identification of the anatomic landmarks is achieved, the CT-free navigation may be sufficient for proper component alignment.

Osteoarthritis with genu valgus deformity has been classified into three types by Ranawat et al. [45]. In this investigation, we focused on type II genu valgus deformity (defined as MA ≥10° valgus based on hip-to-ankle standing radiography), which is relatively rare. This type has a more substantial deformity with medial soft tissue stretching. Surgery is technically demanding because it may be associated with hypoplasia of the distal femur, rotational deformity of the tibia and femur, and maltracking of the patella and other osseous abnormalities along with the soft tissue contracture [46]. There is a greater risk of component malposition, elevation of the joint line, and unplanned conversion to a varus-valgus-constrained type of prosthesis [22, 45, 46]. Deficiencies of the lateral femoral condyle often render the posterior condylar axis improper as a reference for determining femoral component rotation. In order to obtain optimal rotational alignment, the surgeon should repeatedly check by simultaneously using the transepicondylar axis, Whiteside’s line, the posterior condylar line, and the tibial cutting plane. In this study, the tourniquet time was similar between group C and group D. We speculate that intraoperative repeat assessment of femoral component position using multiple reference lines might be the reason for the longer tourniquet time in patients with genu valgum deformity who underwent conventional TKA (Table 2). In this study, CAS-TKAs provided more proper femoral rotational alignment when compared with conventional TKA. A mistake in visual judgment of the chamfer block in the axial plane may be the confounding factor resulting in improper femoral rotation alignment. Although the same surgeon determined the same anatomic landmarks used in both techniques during surgery, to exactly place the chamfer block is difficult using commercial instrumentation when osseous deficiency is present. A mistake in femoral rotation may be difficult to prevent based on visual observation alone. In CAS-TKA, care is taken for identification of anatomic landmarks, which are then sequentially registered into the navigation system. Instead of visual intraoperative judgment of the chamfer block in the axial, the surgeon can judge the accuracy of the chamfer block by taking advantage of the quantitative feedback of the navigation system.

Several limitations of this study should be acknowledged. First, this was a radiographic and CT study, and we were unable to assess the correlations between more proper alignment and long-term functional outcome. Second, this was a retrospective study with all inherent limitations and bias. A single experienced surgeon using the same protocol, which decreases some of the confounding factors, managed all patients. However, the fact that only one surgeon performed all CAS-TKAs is another limitation. Third, only 90 knees with preoperative MA ≥ 15° varus and 54 knees with preoperative MA ≥ 10° valgus were studied. However, it would be difficult to perform a randomized controlled trial comparing CAS-TKA to conventional TKA because of the relative rarity of these deformities in patients undergoing TKA. Fourth, the current study focused on measured resection TKAs with or without assistance in navigation. There were no patients treated using a gap balancing technique; thus, we are unable to comment on whether CAS-TKA would have the similar advantage in this case. Finally, when performing TKA in patients with advanced deformities of the knee, an intramedullary tibial guide can be advantageous. In this study, however, all conventional TKAs and CAS-TKAs were performed with extramedullary tibial guidance.

## Conclusions

In conclusion, CAS-TKA improved the rotational alignment of the femoral component in the advanced valgus knee. Rotational malalignment of the TKA component in the valgus knee occurred in 25 % of the cases using conventional instrumentation, and it could be improved to 7.7 % by CAS-TKA. CAS-TKA appears to be an effective method to properly provide rotational alignment of the femoral component in patients with advanced genu valgum deformity.

## Abbreviations

CAS-TKA: computer-assisted surgery total knee arthroplasty
FF: frontal femoral
FR: femoral rotational
FT: frontal tibial
MA: mechanical axis
SF: sagittal femoral
ST: sagittal tibial
TKA: total knee arthroplasty
