# Erratum to: Improved femoral component rotation in advanced genu valgum deformity using computer-assisted measured resection total knee arthroplasty

**DOI:** 10.1186/s13018-015-0293-6

**Published:** 2015-09-22

**Authors:** Shih-Jie Lin, Chien-Ying Lee, Kuo-Chin Huang, Kuo-Ti Peng, Tsan-Wen Huang, Mel S. Lee, Robert Wen-Wei Hsu, Wun-Jer Shen

**Affiliations:** Department of Orthopaedic Surgery, Chang Gung Memorial Hospital, Chiayi, 6, West Section, Chia-Pu Road, Pu-Tz City, 613 Chia-Yi Hsien Taiwan; Department of Orthopaedic Surgery, Kaohsiung Chang Gung Memorial Hospital, No. 123, DAPI Rd. Niaosng Dist., Kaohsiung City, 83301 Taiwan; Chang Gung University, 259 Wen-Hwa 1st Road, Kwei-Shan, Tao-Yuan, 333 Taiwan; Po-Cheng Orthopedic Institute, 100 Bo-ai, 2nd Road, Zuoying District, Kaohsiung, Taiwan

## Erratum

After publication of the original article [1] it came to our attention that there were errors in Table 3. The data in the first parameter [preoperative coronal MA(°)] was incorrect and has been corrected in Table 3 below.

**Table 3:** Radiographic data of patients with genu varum

	Group A	Group B	
	*n* = 39	*n* = 51	
Parameters	Mean ± SD (min-max)	Mean ± SD (min-max)	*p*-value
Preoperative coronal MA (°)	159.7 ± 3.9 (150–165)	158.3 ± 4.2 (143–165)	0.443
Postoperative coronal MA (°)	179.5 ± 1.7 (178–184)	177.9 ± 2.4 (176–181)	0.572
Frontal femoral angle (°)	90.6 ± 1.7 (86–91)	89.7 ± 1.5 (84–91)	0.414
Sagittal femoral angle (°)	1.6 ± 1.2 (0–6)	3.5 ± 2.8 (1–7)	<0.001*
Femoral rotation angle (°)	1.0 ± 0.7 (0–3)	1.7 ± 1.1 (0–6)	0.127
Frontal tibial angle (°)	90.0 ± 0.5 (89–91)	89.9 ± 1.4 (88–91)	0.714
Sagittal tibial angle (°)	87.9 ± 1.8 (84–90)	87.7 ± 2.2 (80–91)	0.518

Group A: osteoarthritis with genu varum and underwent computer-assisted surgery total knee arthroplasty (CAS-TKA)

Group B: osteoarthritis with genu varum and underwent conventional TKA

*p* values for between-group comparison were determined by Mann–Whitney *U* test

*Statistically significant (*p* < 0.05)
